# Solvent‐free Electrode Fabrication for Next‐generation Batteries: Inception or an Endgame?

**DOI:** 10.1002/chem.202501487

**Published:** 2025-06-18

**Authors:** Milan K. Sadan, Anupriya K Haridas

**Affiliations:** ^1^ Dyson School of Design Engineering Imperial College London South Kensington SW7 2DB UK; ^2^ Warwick Manufacturing Group University of Warwick Coventry CV4 7AL UK

**Keywords:** battery manufacturing, dry electrode fabrication, electrodes, solvent‐free fabrication, sustainable manufacturing

## Abstract

Solvent‐free electrode fabrication (SEF) is a revolutionary advancement in the field of battery manufacturing, providing a compelling, solvent‐free alternative to conventional slurry‐based processing. SEF offers significant benefits, including reduced energy consumption resulting from the elimination of toxic solvents, solvent recovery steps, a lower environmental impact, overall cost reduction, and overcoming of binder migration, encountered during the fabrication of thicker electrodes. However, the practical application of SEF must overcome key challenges, such as achieving uniform particle distribution, ensuring strong electrode cohesion and adhesion, and scaling up to meet industrial production demands. This review provides a comprehensive assessment of the current landscape in dry electrode fabrication technologies, featuring advanced techniques such as dry pressing, spray dry deposition, and 3D printing. The fundamental chemistry of materials in addition to the mechanical, and electrochemical factors critical for optimizing battery performance is elaborated with a clear focus on lithium‐ion batteries (LIBs) from lab to manufacturing stage. Moreover, the article highlights significant recent advancements, identifies critical technical barriers, and outlines imperative research directions necessary to unleash the full potential of dry electrode manufacturing in the realm of sustainable energy storage.

## Graphical abstract

The challenges and opportunities of the emerging solvent‐free electrode fabrication method are discussed in the conceptual review considering the aspects of environmental impact, cost of production, and the resultant energy density.

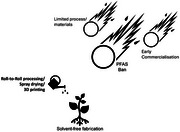

## 1 Introduction

Batteries have become essential in our daily lives, powering everything from personal gadgets to electric vehicles. As nations implement ambitious net‐zero policies, the demand for renewable energy sources is surging, underlining the urgent need for developing efficient next‐generation battery technologies to support the energy transition.^[^ ^1^ ^]^ As a result, the production of lithium has seen remarkable growth over the recent years and has prompted the establishment of gigafactories worldwide.^[^ ^2^ ^]^ In the past decade, the global shipments of lithium‐ion batteries (LIBs) have skyrocketed from 100.8 GWh to an impressive 1202.6 GWh, achieving an extraordinary annual growth rate of 32.4%.^[^ ^2^ ^]^ Nevertheless, it is vital to enhance the performance of the current state‐of‐the‐art LIBs in terms of energy density, cycle life, and safety, to fully realize its potential for various applications. ^[^ ^3^ ^]^ From early on, the advancement of battery technology has been primarily centred on the innovation of new materials to boost the energy density, while the significance of battery manufacturing research is often overlooked.^[^ ^4^ ^]^ In reality, the performance and cost‐efficiency of next‐generation LIBs can be dramatically improved by optimizing the various manufacturing parameters and carefully selecting suitable materials throughout the production process.^[^ ^5^ ^]^ Prioritizing these areas could unlock the full potential of the LIB technology in terms of cost of production and environmental impact.

The current state‐of‐the‐art large‐scale battery manufacturing relies heavily on slurry‐based electrode production, which requires an organic solvent‐assisted processing method for the cathode and a water‐assisted processing method for the anode.^[^ ^6^ ^]^ Although solvent‐based electrode processing techniques produce high‐quality electrodes and facilitate smooth ion diffusion, they come with significant drawbacks. These methods are resource‐intensive, costly, and detrimental to the environment.^[^ ^4^, ^5^, ^6^, ^7^ ^]^ Therefore, it is crucial to explore alternative electrode processing strategies which are facile, affordable and environmentally benign. Solvent‐free electrode fabrication (SEF) is an emerging field that leverages a sustainable way of manufacturing next‐generation battery electrodes without employing any solvents as opposed to the conventional slurry‐based electrode processing.

While the conventional slurry‐based electrode processing limits the thickness of the prepared electrodes (<100 µm), this promising technology enables the fabrication of thicker electrodes (up to the mm range) and improves the energy density.^[^ ^4c^ ^]^ Tesla's adaptation of this promising manufacturing method, pioneered by Maxwell Technologies has revolutionized the global battery manufacturing industry in recent years.^[^ ^4c^ ^]^

Herein, a conceptual evaluation of the emerging dry electrode fabrication processes for LIBs, emphasizing its relevance compared to the state‐of‐the‐art solvent‐mediated electrode processing along with a detailed fundamental understanding of the chemistry involved in the process, basic types of SEF methods and advantages in its implementation from lab‐level to manufacturing‐level are discussed. Finally, an outlook on the prospective research directions and challenges, along with possible mitigation strategies in this area, is outlined, aiming at the rapid development of sustainable LIB manufacturing during the ongoing energy transition.

## 2 Solvent‐free Electrode Fabrication Technique

SEF method mainly focus on the eradicating the organic solvent in the conventional electrode fabrication technique presents an excellent opportunity, offering numerous advantages, including the following.

**Figure 1:**
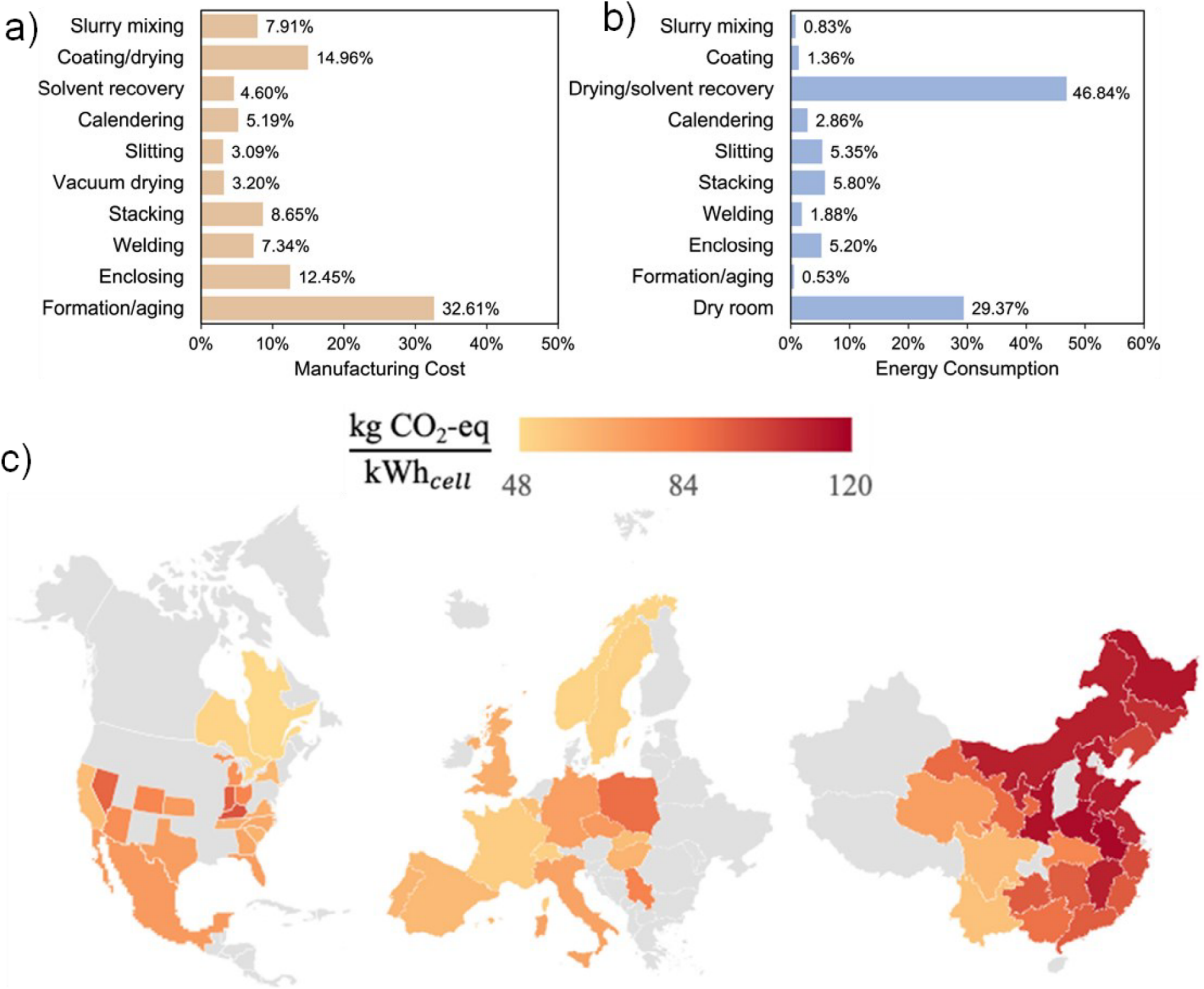
Breakdown of a) manufacturing cost and b) energy consumption associated with conventional solvent‐mediated LIB manufacturing. Reproduced with permission from ^[^ ^5^ ^]^ Copyright 2021, Elsevier, c) Average cradle‐to‐gate carbon footprint of LIB production in various battery manufacturing locations (North America, Europe, and China). Reproduced with permission from ^[^ ^8^ ^]^ Copyright 2024, Elsevier.

**Economic Impact**: It is estimated that the overall cost for coating, drying and solvent recovery involves 19.56% of the total cost (Figure 1a) of the electrode manufacturing ^[^ ^5^ ^]^ and with the additional steps of cell formation, and aging it reaches up to total of 48% of the entire manufacturing cost. ^[^ ^9^ ^]^ Since the drying step and solvent recovery is absent, a significant cost reduction can be achieved.^[^ ^9^ ^]^ Thus, the capital expenditure and operational expenditure can be lowered. SEF can also lead to thicker electrodes; it is already a proven fact that increasing the thickness of electrode to 300 µm and beyond leads to 30–40% decrease in cell level cost ^[^ ^10a, b^ ^]^.

**Environmental Impact**: For the SEF method, 37% less energy consumption is estimated compared to the traditional slurry cast method (Figure 1b). The Carbon dioxide (CO_2_) Footprint (CF) of battery manufacturing is closely linked to the electrode processing method as it constitutes almost 50% of energy consumption owing to the solvent drying/recovery stage (Figure 1c). Additionally, the Global Warming Potential (GWP) for SEF is 49.3 kg CO_2_‐eq per kWh which is 15.1% less than that of the conventional solvent‐mediated electrode manufacturing method.^[^ ^11^ ^]^ Various countries have been making policies that insist on green protocols for industrial processes; for instance, the European Union (EU) Battery Regulation and the US Inflation Reduction Act have already taken necessary steps to reduce the CF in battery production.^[^ ^12^ ^]^ The SEF method for battery manufacturing aligns with the prospective environmental policy regulations and opens avenues for further CF reduction via relevant process optimizations.

**Binder Migration**: Even though binder in an electrode is an inactive material, it plays a crucial role in electrochemical performance especially cycling performance as binders play a significant role in the mechanical strength of the electrodes.^[^ ^13^ ^]^ During the drying process after slurry coating, solvent evaporation occurs due to capillary forces that lead to the binders moving toward the electrode surface. This migration can result in uneven binder distribution along the longitudinal direction, which can significantly impact the performance and efficiency of the final product.^[^ ^14^ ^]^ In a traditional slurry‐based electrode fabrication technique, binder migration is a common phenomenon which becomes predominant in thicker electrodes. The binder migration results in poor adhesion between current collectors and the active materials and can increase contact resistance.^[^ ^15^ ^]^ Additionally, the uneven binder distribution leads to an increase in tortuosity which leads to a decrease in the electrochemical performance of the batteries. The prolonged cycling of the electrode could lead to delamination of the electrode from the current collector.^[^ ^14^ ^]^ Since the SEF method does not involve any solvent, the critical issue associated with binder migration can be avoided, thereby yielding thick electrodes with high performance.

Based on the constituent binders and the processing methods involved, the SEF techniques can be categorized into the following.

### 2.1 Solvent‐free Electrode Fabrication by Dry Pressing/ Roll‐to‐Roll Processing

**Figure 2:**
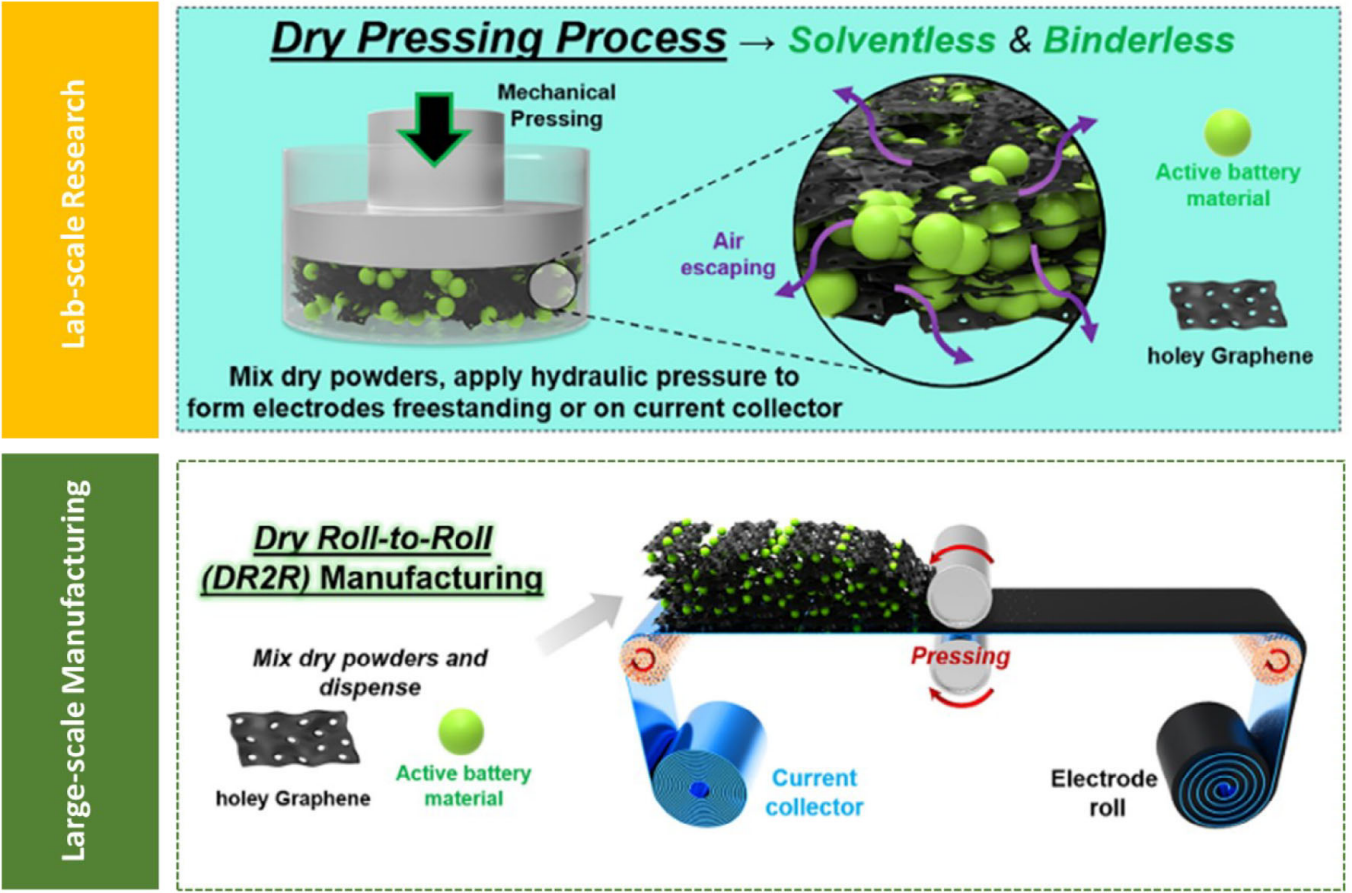
Schematic diagram comparing SEF method via dry pressing technique and roll‐to‐roll manufacturing. Reproduced with permission from.^[^ ^16^ ^]^ Copyright 2019, American Chemical Society.

Dry pressing is a simple SEF method where powdered active materials, conductive additives, and binders are mixed and directly pressed into a current collector using pressure and temperature without the use of toxic solvents. Mostly, this method is relevant to be applied in lab‐scale processing of electrodes as there are significant challenges involved with achieving cohesion of active particles, breaking of active materials and electrode structure with an increase in thickness. Whereas roll‐to‐roll processing of dry electrodes via SEF is an industrially relevant hybrid method of continuous electrode fabrication process and is a modification of the existing slurry‐based electrode fabrication with roll‐to‐roll setup. In this method, a dry powder mixture consisting of the active material, binder and conductive additive is formed into a cohesive and freestanding electrode laminated onto a current collector foil using mechanical compaction, employing a conventional roll‐to‐roll setup at a large‐scale manufacturing level, avoiding the solvents and drying steps. Figure 2 shows a schematic of both the methods of SEF techniques.

#### 2.1.1 Holey Graphene‐assisted Method

**Figure 3:**
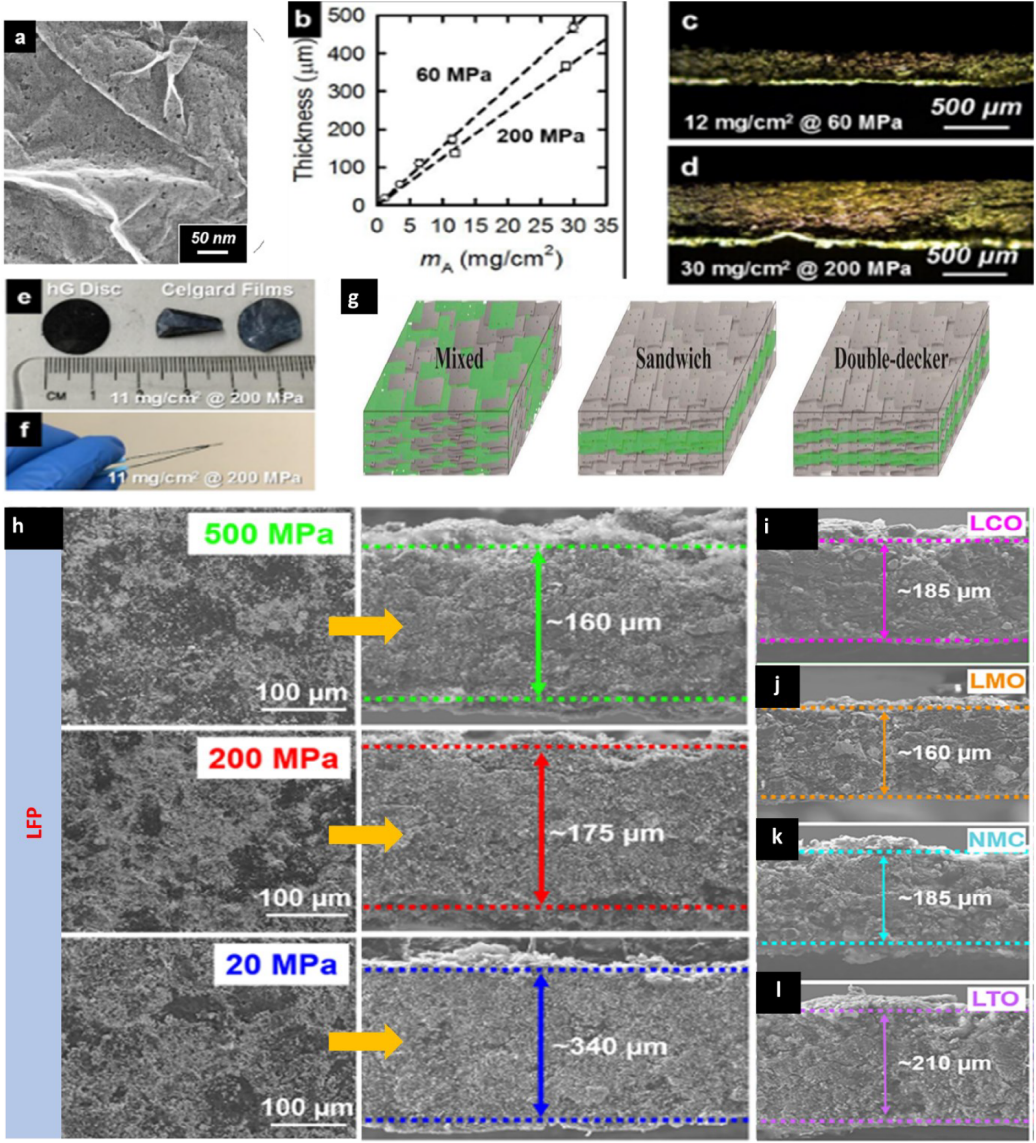
a) TEM image of HG, b) thickness versus areal loading achieved in dry pressed electrodes with HG at various applied pressures, c) and d) the corresponding electrode cross‐sections e) and f) represents the photographic images of the high loading (11 mg cm ^− 2^) dry pressed HG at 200 MPa. Reproduced with permission from ^[^ ^18^ ^]^ Copyright 2019, American Chemical Society. g) Various types of dry‐pressed electrode architectures. Reproduced with permission from ^[^ ^20^ ^]^ Copyright 2017, Elsevier. h) SEM images of LiFePO4 electrodes prepared at varying the applied pressures and the corresponding electrode cross‐sections revealing the variation in electrode thickness, i) electrode cross‐sections of dry pressed HG‐containing i) LCO, j) LMO, k) NMC and l) LTO electrodes prepared at 500 MPa. Reproduced with permission from ^[^ ^16^ ^]^ Copyright 2019, American Chemical Society.

Holey Graphene (HG), the versatile and promising graphene derivative with in‐plane nano‐perforations or holes in the basal plane of the graphene structure (see Figure 3a) has found immense applications in water desalination, gas sensing, electrochemical energy storage and conversion owing to its unique physical (high density‐1.4 g cm^−3^; specific mechanical strength‐18 MPa cm^3^ g^−1^, and electrical (130 S cm^−1^) properties.^[^ ^17^ ^]^ Though over the years it has been used as both a conductive additive and electrode material in dye‐sensitized solar cells, batteries and supercapacitors, they are now being successfully implemented as a component for SEF method. The limited restacking of the graphene nanolayers and the moldability of this porous graphene to various architectures are explored in this process to prepare solvent‐free electrodes. This is possible because the gas molecules trapped within the HG sheets can permeate and escape easily due to the nano perforations, leading to compact monolith formation upon release, whereas in case of pristine graphene, the gas molecules get trapped between the graphene layers during the pressing stage, resulting in loose powder formation upon release.^[^ ^17b^ ^]^ Generally, HG as such or in combination with the active electrode material is dry pressed at ambient pressure without the conventional solvents and binder to generate additive‐free, thick and freestanding, dry electrodes at room temperature. For example, Lin et al. fabricated ultrathick monolithic electrodes of HG with high aerial mass loadings of 12 and 30 mg cm^−2^ (Figure 3b) using a hydraulic dry press by varying the applied pressure and the corresponding electrode cross sections are shown in Figure 3c and d.^[^ ^18^ ^]^ However, the pristine graphene sheets processed under similar compression conditions could not be moulded into mechanically robust discs. As opposed to the normal thick electrodes, wherein the performances degrade with increasing electrode thickness, the low tortuosity levels achieved in HG‐derived thick electrodes contribute to stable and high electrochemical performances. Such dry processed HG electrodes when directly employed in supercapacitors yielded stable gravimetric capacitances even at high current densities up to 30 mA cm^−2^. Alternatively, they are also reported to display ultrahigh areal capacity (∼40 mAh cm^−2^) in Li‐O_2_ batteries indicating promising prospects for practical applications.^[^ ^19^ ^]^ Various electrode architectures, mixed, sandwiched, double decker type (Figure 3g) can be prepared by either mixing with other components or sequentially loading various powdery components prior to compression.^[^ ^20^ ^]^ In a pioneering approach, Hu et al. demonstrated the dry processing of incompressible noncarbon‐based battery materials in combination with HG.^[^ ^16^ ^]^ Freestanding composite electrodes of LiFePO_4_ were prepared with HG in a 1:1 ratio, moulded at various pressures (20 MPa to 500 MPa) without any structural changes, ensuring mechanical robustness. Even though the applied pressure resulted in differences in electrode thickness (see Figure 3h; low pressure leading to thicker electrode formation), it was found that the applied pressure does not impact the electrochemical performances when tested in LIBs. The method was also extended to other electrochemically active materials like LCO, NCM, LMO and LTO at the optimized higher applied pressure of 500 MPa to obtain freestanding solvent‐free electrodes. However, the cross‐sectional SEM images of the electrodes (Figure 3i–l) revealed varying thicknesses ranging from 160–210 µm for the different electrochemically active materials denoting that even at the uniform applied pressures, the obtained electrode

High energy density, conversion‐based cathode, sulfur (S); with low conductivity, have also been prepared employing a similar method retaining almost 80% S in electrode (20 mg electrode) in less than 15 minutes, yielding excellent areal (20 mAh cm^−2^) and volumetric (1787 mAh cm^−3^) capacities which are the highest reported till date and is promising for practical implementation.^[^ ^21^ ^]^

#### 2.1.2 Fibrillation‐Based Method with Polymeric Binders

Fibrillation‐based dry pressing/roll‐roll coating is one of the most investigated methods for commercial level SEF in the recent years. The fibrils (or fibres; often used interchangeably) are continuous and long arrangement of short, thin elongated particles with high aspect ratio. In this straightforward method which can be upscaled to manufacturing stage easily, the active materials together with certain polymeric binder materials (e.g., Polytetrafluoroethylene; PTFE, copolymers of tetrafluoroethylene and other monomers such as ethylene, hexafluoropropylene) form fibrils during the dry mixing and pressing/roll‐roll coating leading to dry film formation.^[^ ^22^ ^]^

**Figure 4:**
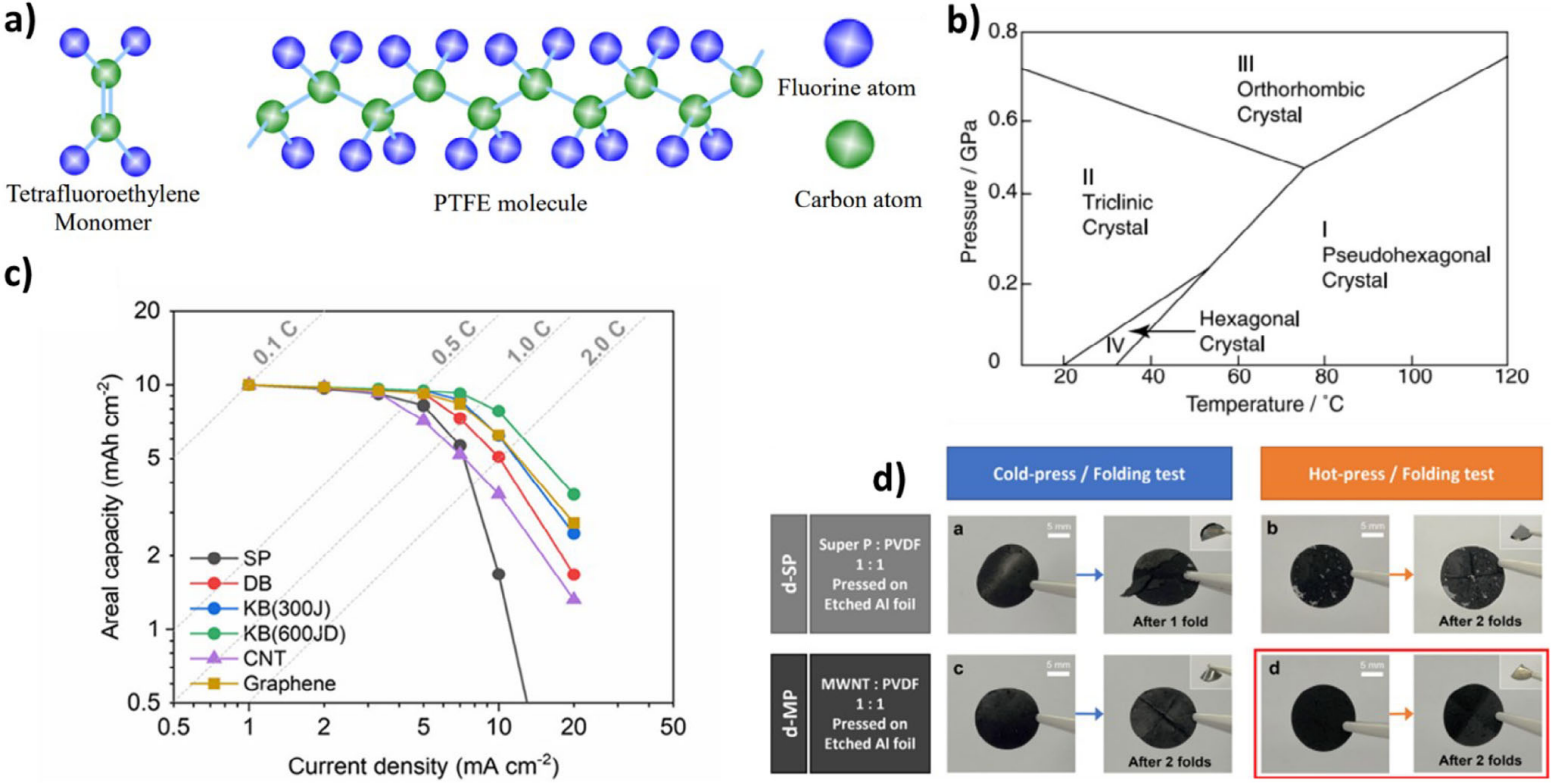
a) Chemical structure of PTFE. Reproduced with permission from ^[^ ^23^ ^]^ Copyright 2017, University of British Columbia. b) Pressure‐temperature phase diagram of PTFE. Reproduced with permission from ^[^ ^24^ ^]^ Copyright 1989, Wiley. c) areal capacity versus Current density profile of various dry pressed NMC electrodes prepared with various conductive additives. Reproduced with permission from ^[^ ^25^ ^]^ Copyright 2025, Royal Society of Chemistry. d) Comparison of dry press‐coating via cold press and hot press. Reproduced with permission from ^[^ ^26^ ^]^ Copyright 2023, Springer Nature.

PTFE is a nonpolar, nonreactive, and thermally stable polymer and is composed of a long straight carbon backbone surrounded by evenly distributed fluorine atoms as shown in Figure 4a.^[^ ^23^ ^]^ At 19 °C, PTFE molecular chain segment changes from 3D order to a less ordered structure (See Figure 4b).^[^ ^24^ ^]^ Beyond 19 °C, PTFE molecule undergoes an untwisting from 180° twist per CF_2_ group to 180 ° twist per 15 groups, resulting in a volume increase by 1.3%.^[^ ^27^ ^]^ At this temperature, the molecular chain of PTFE will become soft, and a small shearing force can pull out the molecular chains on the polymer surface which are called fibrils.^[^ ^28^ ^]^

The second important temperature appears at 30 °C at which twists per 15 groups remain the same; however, the degree of disorder of the rotational orientation of molecules will take place along the long axis at a faster rate which will further increase the volume by 0.5%.^[^ ^29^ ^]^ The higher the temperature, the lower the shear force required for binder fibrillation. On the one hand, polymers with larger molecular weights are not easy to crystallize and easy to fibrillate when preparing polymer powders. On the other hand, after fibrillation, the larger the polymer's molecular weight, the longer the fibril can be formed. Therefore, fibrils and powder particles are more easily entangled with each other to fix the powder particles. When a binder with an average molecular weight of more than 5 million is used, the proportion of binder/activated carbon can be adjusted respectively to maintain the same quality of film formation.^[^ ^22^ ^]^ In conclusion, two requirements necessary for fibrillar crystallization are a high molecular weight polymer and a process to elongate the polymer molecules before crystallization. Other factors, such as temperature, molecular weight distribution, solution concentration, and elongation type and rate, also affect fibrillar crystallization and can be optimized for processing. As the carbon binder domain in thick electrodes is crucial to achieve long lasting batteries with high areal capacities, PTFE fibrillation using carbon nanotubes (CNT) or carbon black additives mixed with various electrochemically active materials (e.g., LFP, NMC, LMO, Graphite) have been investigated in LIBs.^[^ ^25^, ^30^ ^]^ It has been found that unlike the case of solvent assisted electrode processing where delamination of electrode and high electrode resistance are prevalent due to uneven carbon‐binder domain formation, dry pressing NMC and graphite with PTFE binder mixed with conductive additives of good porosity (e.g., ketjen black) are ideal for preparing thick electrodes due to the better distribution of carbon‐binder binder domain achievable with demonstrated areal capacities and composite density extending up to 20 mAh cm^−2^ and 3.65 g cm ^−3^ (See Figure 4c).^[^ ^25^ ^]^ Additionally, the melt viscosity of PTFE is high and stable at temperatures and pressures of 380 °C at 10 GPa, making it versatile and easy to process even using extrusion‐based methods.^[^ ^31a^ ^]^ In a pioneering attempt, Schmidt et al. employed sericin, a biodegradable polypeptide as a nonfluorinated fibrillating binder for sulfur cathodes.^[^ ^31b^ ^]^ The fibrils formed by the shearing of sericin are reported to be shorter and thinner than those of PTFE, offering better connectivity to the nanosized active materials and single carbon particles. Though the dry processed electrodes with sericin demonstrated better wettability and lower overpotential compared to that of PTFE‐based electrodes, no significant improvements were achieved in the capacity and cycle life of the lithium sulfur cells. However, sericin could be a viable alternative to PTFE for the SEF processing of both cathode and anode materials owing to its good electrochemical stability at both higher and lower electrochemical potentials, unlike that of PTFE.^[^ ^31c^ ^]^

#### 2.1.3 Nonfibrillation‐based Method with Other Polymers

Various other polymers which do not form fibrils have been employed to prepare thick electrodes via dry pressing and roll‐to‐roll coating process. In this method, the optimal molecular weight of the polymer, pressure and temperature are critical to achieve the desired freestanding dry‐pressed electrodes. For example, a heated press was used to prepare LFP electrodes with polyvinylidene fluoride (PVDF) binder by applying a temperature and pressure of at 185 °C and 278 psi for 10 minutes.^[^ ^32^ ^]^ Such high levels of compression are required to ensure adequate adhesion to the current collector. While comparing dry pressed NMC electrodes (17.6 mAh cm^−2^) prepared with NMC, PVDF and MWCNT powder mixture pressed onto an etched Al foil by both cold‐pressing (25 °C) and hot‐pressing (180 °C) at an equal pressure of 10 MPa for 30 s (Figure 4d) revealed better adhesion of the powders on to the current collector in the latter method. This was attributed to the temperature being above the melting point of PVDF (177 °C) initiating thermal activation of the binder producing a binding effect.^[^ ^26^ ^]^ Hun et al. prepared dry pressed electrodes by applying a pressure of 500 kg on polyacrylonitrile‐sulfur powder mixture and subjected it to a simple heating process to generate binder and conductive agent‐free sulfurized polyacrylonitrile cathode disk to directly use as electrodes.^[^ ^33a^ ^]^ The cost‐effective and commercially available aliphatic polycarbonate; poly(lactide‐co‐propylene carbonate), was demonstrated as a functional binder for SEF method recently via hot pressing to prepare LiFePO_4_ electrodes. The prepared electrodes with an areal loading of 57 mg cm^−2^ exhibited a competent capacity of 135.5 mAh g^−1^ at 0.2C.^[^ ^33b^ ^]^ Such attempts to employ functional, nonfluorinated binders have huge potential to be extended to the SEF processing of other cathode and anode materials and can sustainably enhance the energy density of next‐generation batteries.

#### 2.1.4 Spray Drying

**Figure 5:**
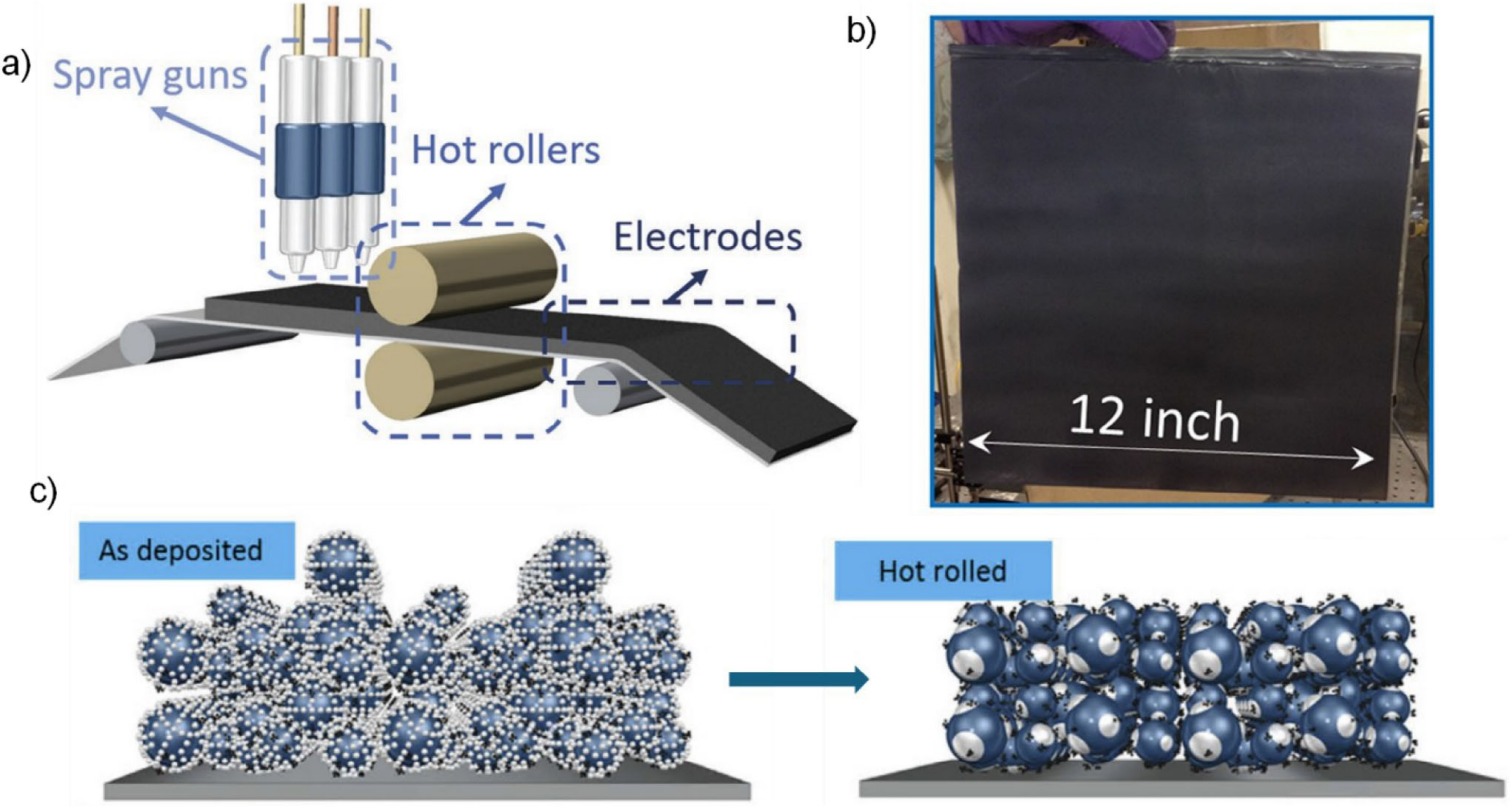
a) Schematic diagram of the dry printing process. Reproduced with permission from ^[^ ^37^ ^]^ Copyright 2017, Wiley., b) photographic image of the spray dried electrode and c) 3D representation of the spray dried electrode before and after hot rolling. Reproduced with permission from ^[^ ^35a^ ^]^ Copyright 2016, Springer Nature.

Spray drying with dry powder is an exceptionally adaptable method for producing SEF, allowing for scalable operations without the need for large equipment.^[^ ^34^ ^]^ This process begins with the preparation of a dry powder mixture, which combines active materials, a binder, and conductive carbon. By applying a high voltage, typically between 30 and 40 kV, to the mixture, a cloud of charged particles are created.^[^ ^35^ ^]^ These particles are then directed toward a grounded current collector with the help of an air supply, ensuring a uniform coating as shown in Figure 5a. The spray drying is highly scalable, as can be seen in Figure 5b. To finalize the process, the coated material on the current collector undergoes hot pressing, which thermally activates (Figure 5c) the binder and guarantees a consistent, high‐quality coating.^[^ ^36^ ^]^ This innovative approach not only streamlines production but also enhances the performance of the materials, making it a compelling choice for advanced applications.

Ludwig et al. reported a spray‐dried LiCoO_2_ cathode electrode using PVDF binder and carbon black. The spray‐dried electrode showed almost twice the bonding performance and better capacity retention compared to the conventional slurry electrode.^[^ ^35a^ ^]^ Zhang et al. optimized the binder content in the spray drying method; when the binder content is too low (5%) or too high (10%), the binder‐conductive agent matrix was found to be agglomerated unevenly in the electrode, which affects the mechanical and electrochemical properties.^[^ ^38^ ^]^ Other factors that affect the electrochemical properties are the morphology of the conductive agents ^[^ ^39^ ^]^, smaller particle size and higher melting point of binders.^[^ ^35b^ ^]^ Apart from the spray drying parameters, the premixing stage and the calendaring step after the spray drying is also important. Lestriez et al. reported that premixing for long time (>17 minutes) result in the reduction in agglomerates and shorter periods (<3 minutes) result in the formation of too many agglomerates in the dry powder.^[^ ^35b^ ^]^ The graphite electrode showed maximum rate performance up to 5 C‐rate and capacity retention of 90% after 150 cycles for the 3 minutes moderated mixed particle. Also, calendaring at a lower temperature than the melting point of the binder is essential to minimize the electrical resistance and tortuosity coefficient of the electrodes.^[^ ^35b^ ^]^

### 2.2 3D Printing

Additive manufacturing or 3D printing is an advanced method of 3D structures sequentially depositing the individual layers. 3D printing offers flexibility in terms of dimension of the electrodes, improved material utilization, engineering a porous structure, while reducing the overall cost of manufacturing.^[^ ^40^ ^]^ Although there have been several types of 3D printing available for electrode fabrication, most of these methods include inks which involve solvents.^[^ ^41^ ^]^ However, methods like Fused Deposition Modelling (FDM) and Selective Laser Sintering (SLS) have the potential for developing solvent‐free electrodes without the use of hazardous per‐and polyfluoroalkyl substances (PFAS) as binders, unlike the previously discussed SEF methods. In FDM (also known as Fused Filament Fabrication), thermoplastic filaments such as polylactic acid (PLA), Acrylonitrile Butadiene Styrene (ABS), Polycarbonate etc. are heated with a resistance heater until they achieve a semi‐molten state. These filaments are then extruded through a small nozzle, enabling precise layer‐by‐layer deposition of material.^[^ ^40^ ^]^ Generally, FDM printers are low‐cost, low‐maintenance, and highly versatile in scalability of prototyping.^[^ ^42^ ^]^

**Figure 6:**
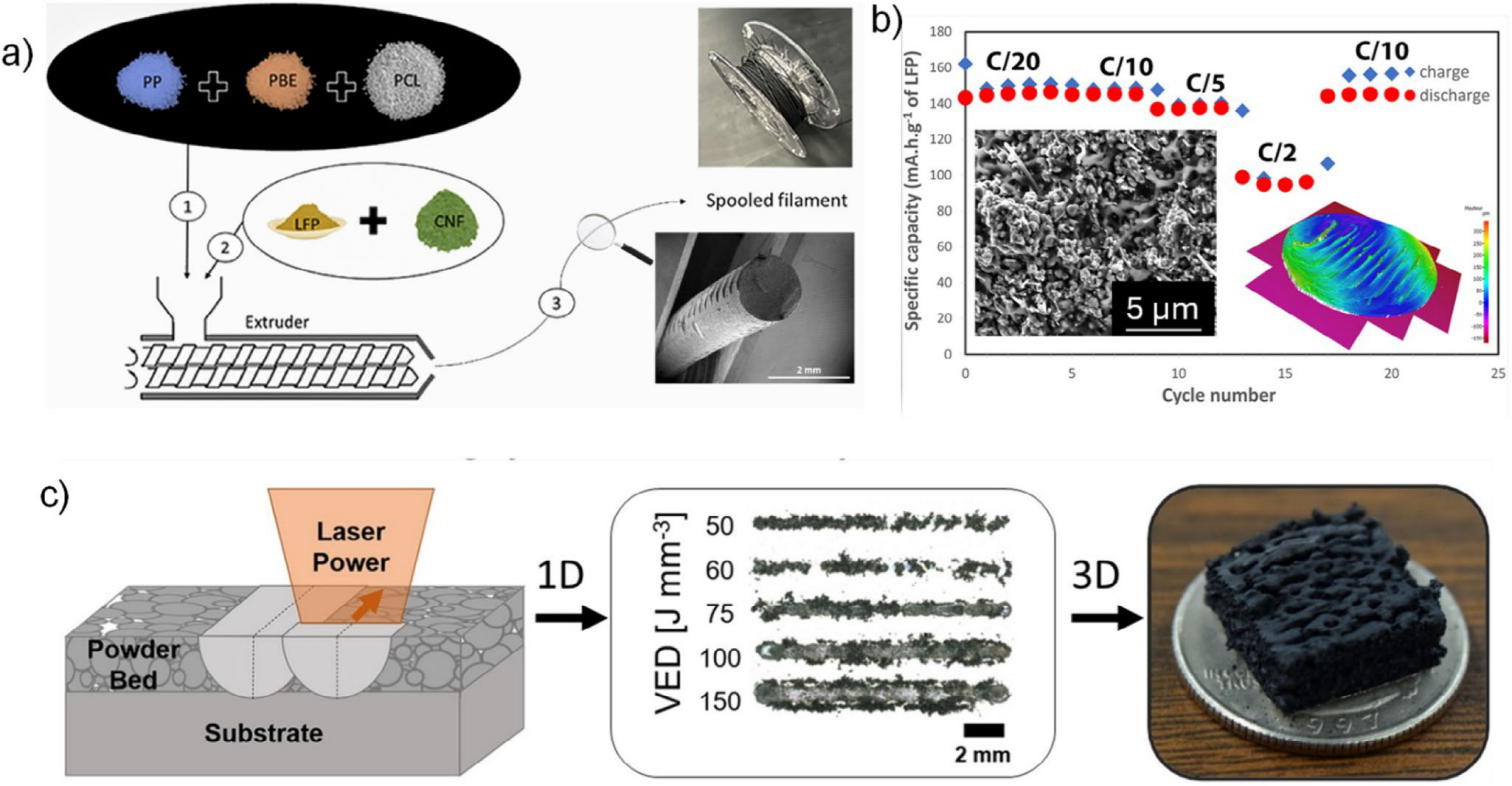
a) Schematic diagram of spooled filament preparation of LiFePO4 electrodes via 3D printing b) rate performance of the 3D printed LiFePO4 cathode at various C rates from C/20 to C/2 (inset: SEM image of 3D printed electrode). Reproduced with permission from ^[^ ^43^ ^]^ Copyright 2024, Elsevier. and c) Schematic diagram of the SLS method and the prepared NCA cathode Reproduced with permission from ^[^ ^44^ ^]^ Copyright 2024, Elsevier.

Recently, there have been several efforts to fabricate battery electrodes by FDM‐based 3D printing; for instance, Golodnitsky et al. reported LiFePO_4_ cathode and Li_4_Ti_5_O_12_ anode for LIBs using PLA with an electrode thickness of 200 µm.^[^ ^45^ ^]^ However, the electrochemical performances were inferior compared to the state‐of‐the‐art reports. Only 50% of the theoretical capacity was obtained owing to the low electrochemically active material content, and further engineering is required in the electrode fabrication to maximize the conductivity of the electrode. Dupont et al. Improvised the printing by using a polymer blend consisting of polycaprolactone and polypropylene to form 200 µm LiFePO_4_ cathodes as shown in Figure 6a.^[^ ^43^ ^]^ The electrochemical properties and fabrication were optimized based on the introduction and optimization of the polymer, polypropylene–block‐ polyethylene‐based elastomer. The LiFePO_4_ half‐cell delivered 85% of the theoretical capacity with a decent rate performance of 82 mAh g^−1^ achieved at 0.5C (C/2) rate as shown in Figure 6b. In SLS, a powerful laser selectively melts areas of a powder bed, constructing a 3D object layer by layer. Unlike slurry‐based 3D printing techniques, SLS enhances active material loading by removing the requirement for binders or solvents in the process. This increased active material loading allows for the creation of 3D electrode structures that achieve higher volumetric energy densities. Julie *et* al. reported LiNi_0.80_Co_0.15_Al_0.05_O_2_ cathode material prepared via the SLS method (Figure 6c) and investigated how the variation of various SLS processing parameters such as laser power and scanning speed affects the morphology, crystalline phases, and internal structure.^[^ ^44^ ^]^

## 3 Summary and Outlook

The various emerging SEF methods are discussed critically evaluating their applicability in next‐generation battery manufacturing methods. The main advantage of the dry pressing/roll‐to‐roll method is the scalability and ease of the process, requiring minimal steps resulting in comparatively less time to fabricate the electrode films. However, a proper dispersion of the carbon binder domain via systematic slurry optimizations is necessary to achieve uniform electrodes with this process to ensure high‐rate performances. Additionally, the initial capital cost for establishing the roll‐to‐roll method setup is high, which limits its wide application. The spray drying process which is a modification of the simple dry pressing/roll‐to‐roll method, requires a high voltage to spread the electrostatically charged polymer solution, raising the capital and operational cost substantially. Spray drying process also leads to a higher carbon footprint than that of the simple dry pressing/roll‐to‐roll method, owing to the resultant higher energy consumption in the overall process. There are limitations associated with the geometry of the electrode films formed with the dry pressing/roll‐to‐roll process (even with spray drying), as only 2D sheets/films can be prepared. The active material rupture/breakage and delamination of the formed electrode films from the current collector during the battery operation is another huge challenge. It is important to carry out micro/macro structural investigations for a deep understanding of in situ microstructural evolution. 3D printing is the most versatile of all the SEF methods, as it can support complex geometries and customized electrode architectures to suit various applications. However, the high capital cost associated with setting up a good resolution printer and the slow process of electrode preparation requiring complex ink formulations are the main drawbacks which limit their commercial applications.

Despite these drawbacks, the commercial exploration of existing SEF methods has outpaced thorough research, particularly in the areas of material selection, process optimization and development stages. This has created enormous challenges regarding the fundamental understanding of the physics and chemistry governing the process. Consequently, gigafactory‐level production of SEFs has yet to be realized. Therefore, extensive research and development are essential, particularly concerning process optimization, material selection, and large‐scale manufacturing to avoid the early commercialization issues. One of the main challenges in the SEF method is the inability to form a cohesive film with certain binders. It is crucial to have fundamental investigations channelled to understand the impact of the molecular weight and branching of the employed binder materials and the relevance of particle size and shape of the electrochemically active materials, in the various SEF methods. Moreover, the potential regulation and restrictions surrounding the use of PFAS and challenges associated with material/method selection also impede the progress of this promising technology. PFAS are fluorinated polymers that repel both water and oil due to their strong carbon‐fluorine (C‐F) bonds.^[^ ^46^ ^]^ The persistence of these polymers in the environment has raised significant concerns about their disposal at the end of life, as well as their accumulation in the human bloodstream, prompting a series of regulatory policies against them.^[^ ^47^ ^]^ Currently, most of the SEF technologies rely heavily on popular PFAS materials such as PTFE or PVDF. The limited choice of binders for SEF poses a significant challenge in the development of this technique and necessitates extensive research to be carried out on alternative binder materials (e.g., biopolymer and ceramic materials) in both academic and industrial settings. Though a few bio‐based functional binders have been reported for SEF, materials like aliphatic polycarbonates, beeswax, lignin and cellulose could be ideal sustainable alternate binders for SEF. The low mechanical stability of the formed films with these biopolymers could be improved by extending the research with composite approaches involving suitable filler materials. Additionally, investigating the fibrillation capability of biopolymers could open more choices of PFAS‐free binders for SEF. The existing challenges in SEF research also open opportunities for exploring extended research areas. For example, there is considerable potential for innovation in SEF by facilitating solventless ceramic‐based 3D printing methods and cost‐effective development of existing materials like HG. Collaborating across multiple disciplines of material science, process engineering, and electrochemistry could lead to innovative solutions for the current challenges faced in SEF methods for battery manufacturing.

## Conflict of Interest

The authors declare no conflict of interest.

## Data Availability

The data that support the findings of this study are available from the corresponding author upon reasonable request.
